# Estimating within-patient sagittal pelvic tilt change from routine anteroposterior hip radiographs in patients undergoing total hip arthroplasty: development and temporal validation

**DOI:** 10.1302/2633-1462.710.BJO-2026-0113.R1

**Published:** 2026-10-08

**Authors:** Kyosuke Kobayashi, Kenichi Kidera, Syuta Yamada, Tatsuya Fukushima, Akira Hozumi

**Affiliations:** 1 Department of Orthopaedic Surgery, Nijigaoka Hospital, Nagasaki, Japan

**Keywords:** Sagittal pelvic tilt, Regression model, total hip arthroplasty, within-patient change, total hip arthroplasty (THA), radiography, hip, pubic symphysis, pelvic tilt, Linear regression analysis, root mean square, primary THA, developmental dysplasia of the hip (DDH)

## Abstract

**Aims:**

Changes in sagittal pelvic tilt influence functional cup orientation in total hip arthroplasty (THA). Although lateral spinopelvic radiographs are the reference standard, they are not routinely obtained, whereas anteroposterior (AP) hip radiographs are widely used. This study evaluated whether within-patient pelvic tilt changes can be accurately estimated using AP radiographs.

**Methods:**

This retrospective observational study included 116 patients who underwent primary THA (derivation cohort). Supine and standing AP radiographs and CT data were acquired preoperatively. CT-derived synthetic radiographs were generated and adjusted to match the pelvic foramen ratio (PFR) measured on AP radiographs, yielding adjusted synthetic (AS) radiographs. For the supine-to-standing transition, the change in anterior pelvic plane tilt (ΔAPPt) measured on AS radiographs was used as the reference outcome, and the change in pubic symphysis-to-transischial line distance (ΔPSTI, mm) on AP radiographs was used as the predictor. A linear regression model was developed to estimate ΔAPPt from ΔPSTI. Internal validation was performed using fivefold cross-validation. Independent temporal validation was performed in a separate cohort (n = 80).

**Results:**

The derived equation was (ΔAPPt = −0.74 × ΔPSTI + 1.2). Fivefold cross-validation showed a mean error of 0.01° (root mean square error (RMSE) 1.4°, mean absolute error (MAE) 1.0°; 95% limits of agreement (LoA) −2.6° to 2.7°). Absolute error was ≤ 3° in 112 out of 116 (96.6%) cases and ≤ 5° in 116 out of 116 (100%). Independent temporal validation showed a mean error of 0.2° (RMSE 1.3°, MAE 0.9°; 95% LoA −2.4° to 2.8°). Absolute error was ≤ 3° in 75 out of 80 cases (93.8%) and ≤ 5° in 80 out of 80 (100%), within an observed ΔAPPt range of −15.3° to 8.0°.

**Conclusion:**

A regression equation based on ΔPSTI estimated supine-to-standing within-patient ΔAPPt from routine AP hip radiographs with a clinically acceptable agreement (95% LoA approximately ± 3°).

Cite this article: *Bone Jt Open* 2026;7(10):1280–1287.

## Introduction

Sagittal pelvic tilt changes with posture and influences functional component orientation in total hip arthroplasty (THA).^1,2^ Excessive posterior pelvic tilt from preoperative supine to postoperative standing has also been associated with an increased risk of anterior instability after THA.^3^ Therefore, assessment and prediction of sagittal pelvic tilt are important for interpreting functional component orientation across postures and after THA.^4,5^

Lateral spinopelvic radiographs are considered the reference standard for evaluating sagittal pelvic parameters, including anterior pelvic plane tilt (APPt). However, they are not routinely obtained during standard care at many institutions.^6,7^

In contrast, anteroposterior (AP) hip radiographs are routinely obtained before and after THA in standard clinical practice. This has prompted interest in estimating sagittal pelvic tilt from AP radiographs using indirect anatomical indicators. Although previous studies have attempted to estimate absolute sagittal pelvic tilt using coronal parameters such as the pelvic foramen ratio (PFR) or the pubic symphysis-to-transischial line distance (PSTI), interindividual anatomical variability fundamentally limits the accuracy of estimating absolute sagittal pelvic tilt from AP radiographs alone.^8-12^ Importantly, several studies have demonstrated strong correlations between changes in sagittal pelvic tilt and changes in these coronal parameters,^9-12^ suggesting that a within-patient, change-based approach may enable reliable assessment of sagittal pelvic tilt change using AP radiographs. EOS-based studies (EOS Imaging, France) have shown that coronal surrogates can capture within-patient changes in sagittal pelvic tilt across specific postural transitions;^12^ however, the extent to which such change-based relationships translate to routine AP hip radiographs (particularly for the supine-to-standing transition) has not been established with independent temporal validation.

Accordingly, we sought to provide a simple, validated change-based estimator of supine-to-standing APPt change using a routinely measurable AP parameter (ΔPSTI). We hypothesized that supine-to-standing ΔAPPt could be estimated with clinically acceptable accuracy from ΔPSTI measured on standard AP radiographs. The aims of this study were to develop a linear regression model to estimate supine-to-standing ΔAPPt using ΔPSTI and to evaluate model accuracy using fivefold cross-validation and independent temporal validation in a separate cohort.

## Methods

This retrospective observational study was approved by the Institutional Review Board of Nijigaoka Hospital (approval number 24004). Written informed consent was waived, and an opt-out procedure was used.

Between April 2024 and March 2025, 126 consecutive patients underwent primary THA at our institution. Patients who were unable to stand or had severe femoral head deformity were excluded (n = 6). Four additional patients were excluded because PFR could not be measured on standing AP radiographs, as marked posterior pelvic tilt obscured the landmarks required to measure PFR. The remaining 116 patients constituted the derivation cohort.

A separate consecutive series of patients who underwent primary THA between April 2025 and December 2025 was identified (n = 84). In total, four patients were excluded because standing radiographs were unavailable (n = 1) or PFR was not measurable owing to the same landmark obscuration (n = 3). The remaining 80 patients constituted the independent temporal validation cohort. Patient characteristics and underlying diagnoses are shown in Table I.

**Table I.** Patient demographic characteristics.

Variable	Derivation cohort	Validation cohort
Patients, n	116	80
Mean age, yrs (range)	69 (40 to 89)	68 (44 to 85)
Mean body weight, kg (range)	57 (32 to 90)	59 (43 to 80)
Mean BMI, kg/m^2^ (range)	24.1 (16.3 to 35.9)	24.4 (17.5 to 32.0)
Females, n (%)	105 (90.5)	71 (88.8)
Primary OA, n (%)	46 (39.7)	20 (25.0)
DDH, n (%)	65 (56.0)	55 (68.8)
ONFH, n (%)	4 (3.5)	5 (6.3)
SIF, n (%)	1 (0.8)	0 (0)

DDH, developmental dysplasia of the hip; OA, osteoarthritis; ONFH, osteonecrosis of the femoral head; SIF, subchondral insufficiency fracture.

### Radiological acquisition and measurements

All radiographs and CT data used for model development and validation were acquired preoperatively. AP hip radiographs were acquired in the supine and standing positions. The focus–object distance was set at 120 cm and the central beam was directed to the upper margin of the pubic symphysis. Coronal parameters (PSTI and PFR) were measured on AP radiographs.

PSTI was defined as the vertical distance from the transischial line to the upper border of the pubic symphysis.^10^

PFR was defined as the ratio of the pelvic foramen vertical diameter to its maximum horizontal diameter (vertical/horizontal).^13^ The vertical diameter was measured between the superior border of the pubic symphysis and the line connecting the most inferior points of the sacroiliac joints. Each parameter was measured three times on each radiograph, and the mean of the three measurements was used for analysis. Radiological measurements were conducted using SYNAPSE OP-A (Fujifilm, Japan).

### Generation and adjustment of synthetic radiographs

Preoperative CT data with a 2 mm slice thickness were used to generate CT-derived digitally reconstructed radiographs (DRRs; synthetic AP and lateral radiographs) based on the supine pelvic orientation at CT acquisition, using ZedHip software (Lexi, Japan).

Adjusted synthetic (AS) radiographs were generated separately for the supine and standing positions by manually adjusting sagittal pelvic tilt on the CT-derived synthetic images until PFR on the synthetic AP radiograph matched that measured on the corresponding clinical AP radiograph for each posture (Figure 1).^14^ Because pelvic orientation at CT acquisition may differ from that during clinical radiography even in the supine position, this PFR-based adjustment was performed for both supine and standing images. Axial and coronal pelvic rotations were kept identical to those of the original CT data. APPt was measured on AS lateral radiographs. PSTI was also measured on AS AP radiographs to assess its association with PSTI measured on clinical AP radiographs.

**Fig. 1:**
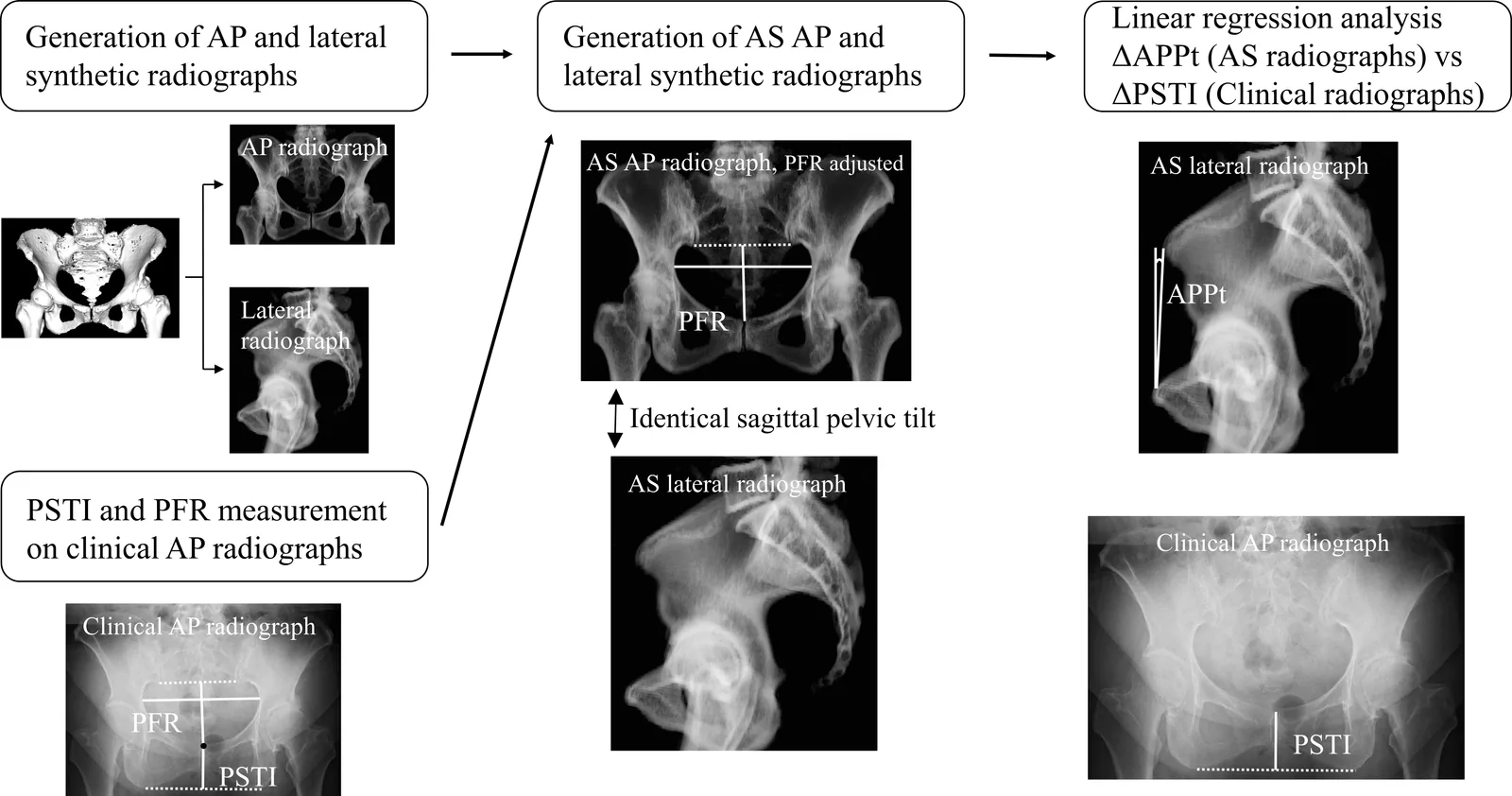
Study workflow and representative images. Preoperative CT data were used to generate synthetic anteroposterior (AP) and lateral radiographs. Pelvic foramen ratio (PFR) and pubic symphysis-to-transischial line distance (PSTI) were measured on clinical AP radiographs. For each posture (supine and standing), CT-derived synthetic AP radiographs were manually adjusted in sagittal pelvic tilt until the PFR matched that on the corresponding clinical AP radiograph, generating adjusted synthetic (AS) AP and lateral radiographs. Anterior pelvic plane tilt (APPt) was measured on AS lateral radiographs, and PSTI was measured on clinical AP radiographs. Supine-to-standing changes (ΔAPPt and ΔPSTI; standing minus supine) were evaluated using linear regression.

APPt was defined as the angle between the line connecting the midpoint of the anterior superior iliac spines and the superior margin of the pubic symphysis and a vertical reference line. Positive APPt indicates anterior pelvic tilt (anterior rotation of the APP relative to the vertical reference line).

For the supine-to-standing transition, changes were defined as: ΔPSTI = PSTI_standing − PSTI_supine and ΔAPPt = APPt_standing − APPt_supine. ΔAPPt served as the reference outcome for model development and validation.

### Supplementary CT-based analysis supporting AS-measured APPt

To support the use of AS-measured APPt as a practical reference outcome, we performed a supplementary CT-based analysis in ten patients. AS-measured APPt was compared with CT-derived posture-specific APPt estimates to assess internal consistency of the PFR-based calibration, not criterion validity against an independent imaging reference standard. Details are provided in the Supplementary Material.

### Regression analysis for estimating changes in APPt using PSTI

We first assessed the association of PSTI measurements between clinical AP radiographs and AS AP radiographs. Subsequently, for the supine-to-standing transition, changes in APPt on AS radiographs (ΔAPPt) and changes in PSTI on clinical AP radiographs (ΔPSTI) were calculated. A linear regression model was used to derive a prediction equation to estimate ΔAPPt from ΔPSTI.

Fivefold cross-validation was performed in the derivation cohort (n = 116), with pooled out-of-fold predictions used to compute accuracy metrics. Patients were randomly assigned to five approximately equal-sized folds, and fold assignment was kept fixed for all analyses. Prediction error was defined as ΔAPPt measured on AS radiographs minus estimated ΔAPPt. Prediction accuracy was quantified using mean error (bias), root mean square error (RMSE), mean absolute error (MAE), and 95% limits of agreement (LoA). In addition, the proportions of cases with prediction errors within ± 3° and ± 5° were calculated.

To evaluate the effect of measurement variation among observers on prediction accuracy, an observer-specific analysis was performed in the 30-patient reliability subset. The primary observer (KKo) generated AS radiographs using observer-specific PFR measurements and measured observed ΔAPPt on the corresponding radiographs. Predicted ΔAPPt was calculated using observer-specific ΔPSTI measurements and the derivation equation. Accuracy was assessed using the same metrics.

Independent temporal validation was performed in a separate consecutive cohort (n = 80). AS radiographs were generated using the same workflow, ΔAPPt and ΔPSTI were calculated, and the derivation equation was applied without re-estimating coefficients. Accuracy was assessed using the same metrics.

Exploratory sub-group analyses were performed to assess model robustness. First, the validation cohort was stratified by AS-measured ΔAPPt (< −10°; −10° to < −5°; −5° to < 0°; and ≥ 0°). Second, acetabular morphology was classified as developmental dysplasia of the hip (DDH) when the lateral centre-edge angle was < 25° or the Sharp’s angle was > 45°; otherwise, it was classified as radiologically normal acetabulum. Radiological parameters were compared between groups in both cohorts, and prediction accuracy was summarized by sub-group in the independent temporal validation cohort.

### Statistical analysis

Among included cases, there were no missing data for variables used in the primary analyses; therefore, no imputation was performed, and analyses were conducted on complete cases. Descriptive statistics were used to summarize the data. Normality was assessed using the Shapiro–Wilk test, as appropriate. Associations between PSTI measured on clinical AP radiographs and PSTI measured on synthetic AS AP radiographs were evaluated using Spearman’s rank correlation coefficient (ρ).

Measurement reliability was assessed using intraclass correlation coefficients (ICCs) with 95% CIs. Intraobserver reliability for APPt and PSTI measurements in the supine and standing positions was assessed in the full derivation cohort (n = 116) using three repeated measurements by the primary observer (KKo) and ICC (3,1), based on a two-way mixed-effects model and absolute agreement. Interobserver reliability for AS APPt, PFR, and PSTI measurements was assessed in a randomly selected subset of 30 patients by three independent observers (KKo, KKi, AH) who were blinded to the original measurements and study results, using ICC (2,1), based on a two-way random-effects model, absolute agreement, and single measurements.

Based on a power analysis for linear regression, a sample size of 55 was required to detect a medium effect size (f² = 0.15) with one predictor, *α* = 0.05, and power = 0.80. Model assumptions were assessed using residual–fitted plots and the Shapiro–Wilk test of residuals.

Statistical analyses were primarily performed using JMP v. 18.0 (SAS Institute, USA). ICC analyses were performed using EZR (Saitama Medical Center, Jichi Medical University, Japan), ^15^ which is a graphical user interface for R (R Foundation for Statistical Computing, Austria) based on R Commander. A two-sided p-value < 0.05 was considered statistically significant.

## Results

In the derivation cohort, APPt on AS radiographs ranged from −26.9° to 15.2° in the supine position and from −29.9° to 15.8° in the standing position. The APPt change measured on AS radiographs for the supine-to-standing transition (ΔAPPt) ranged from −15.6° to 17.1°, and the corresponding change in PSTI (ΔPSTI) ranged from −20.6 mm to 25.7 mm. In the independent temporal validation cohort, ΔAPPt ranged from −15.3° to 8.0°, and ΔPSTI ranged from −8.5 mm to 27.9 mm (Table II). Model development and validation were conducted within these observed ΔAPPt ranges.

**Table II.** Anterior pelvic plane tilt (APPt) measured on adjusted synthetic radiographs and pubic symphysis-to-transischial line distance measured on clinical anteroposterior radiographs.

Variable	Mean derivation cohort (range)	Mean validation cohort (range)
Supine APPt, °	0.4 (−26.9 to 15.2)	2.0 (−24.2 to 25.6)
Standing APPt, °	−1.9 (−29.9 to 15.8)	−1.5 (−31.2 to 22.8)
ΔAPPt, °*	−2.3 (−15.6 to 17.1)	−3.4 (−15.3 to 8.0)
Supine PSTI, mm	46.3 (23.5 to 76.7)	45.6 (23.5 to 78.4)
Standing PSTI, mm	51.0 (26.3 to 88.3)	52.2 (27.7 to 92.3)
ΔPSTI, mm*	4.7 (−20.6 to 25.7)	6.5 (−8.5 to 27.9)

* Δ indicates supine-to-standing change (standing minus supine).

PSTI, pubic symphysis-to-transischial line distance.

PSTI values measured on AS radiographs were strongly correlated with those measured on clinical AP radiographs in both the supine and standing positions (supine: ρ = 0.992; standing: ρ = 0.992; Figure 2). Measurement reliability was excellent. Intraobserver reliability for APPt and PSTI measurements in both the supine and standing positions showed ICCs ranging from 0.993 to 0.999, with corresponding 95% CIs ranging from 0.990 to 0.9992. In the randomly selected 30-patient subset, interobserver reliability was excellent for AS APPt, PSTI, and PFR measurements, with ICC (2,1) values ranging from 0.966 to 0.998 (95% CI 0.880 to 0.999) across positions. Detailed ICC values and 95% CIs are presented in Supplementary Table S1.

**Fig. 2:**
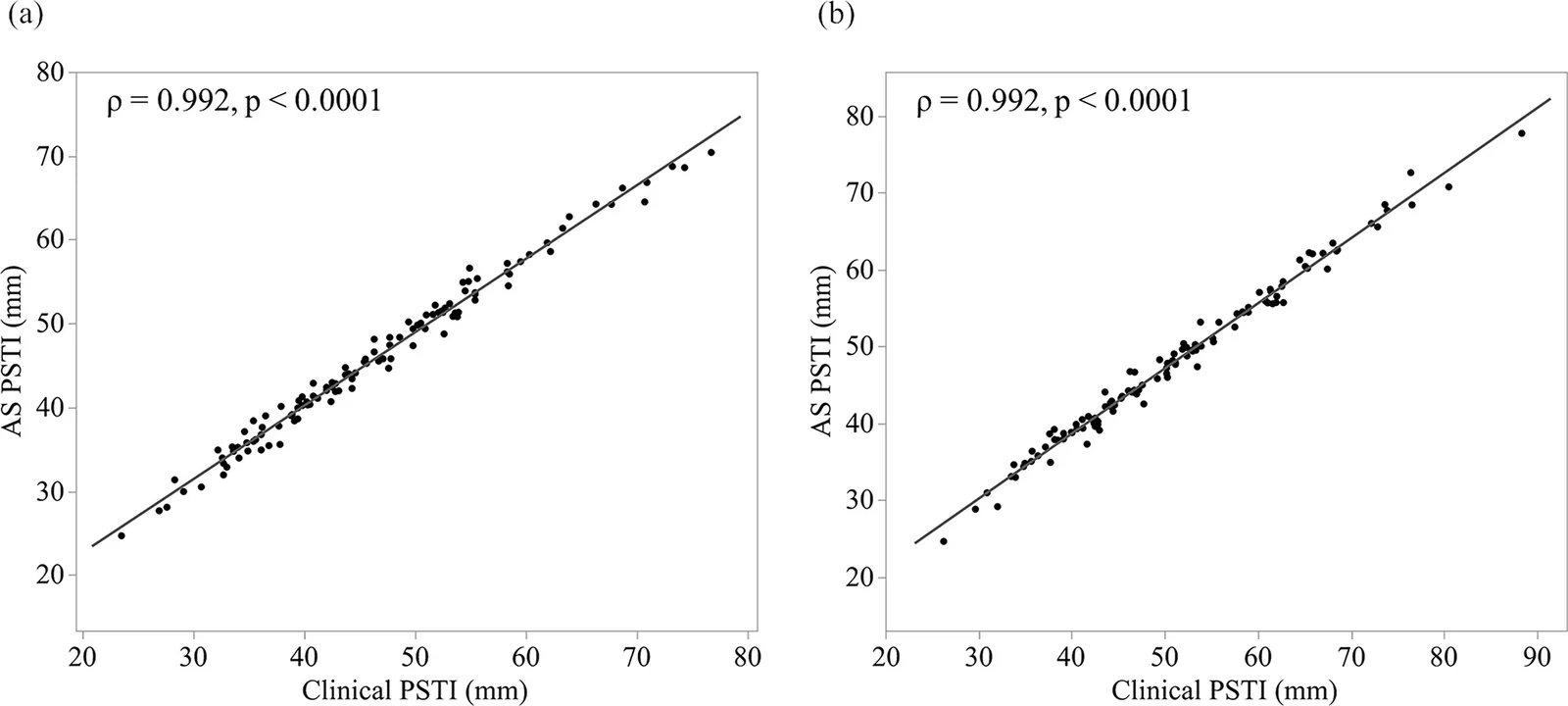
Correlation of pubic symphysis-to-transischial line distance (PSTI) between adjusted synthetic (AS) and clinical anteroposterior radiographs. a) Supine position and b) standing position. Strong correlations were observed in both positions (supine: ρ = 0.992; standing: ρ = 0.992). p-values were calculated using Spearman’s rank correlation.

In the supplementary CT-based analysis of ten patients, AS-measured APPt showed close agreement with CT-derived posture-specific APPt estimates in both the supine and standing positions. The mean bias was −0.4° with 95% LoA from −1.7° to 0.8° in the supine position and −0.3° with 95% LoA from −1.3° to 0.7° in the standing position. Pearson correlation was high in both positions (supine: *r* = 0.995, (95% CI 0.978 to 0.999); standing: *r* = 0.999, (95% CI 0.994 to 0.999)) (Supplementary Table S2 and Supplementary Figure S1).

Linear regression analysis demonstrated a significant negative association between ΔPSTI on clinical AP radiographs and ΔAPPt on AS radiographs (slope = −0.74; (95% CI −0.77 to −0.70); p < 0.001; R² = 0.94), yielding the following equation: ΔAPPt (°) = −0.74 × ΔPSTI (mm) + 1.2 (Figure 3).

**Fig. 3:**
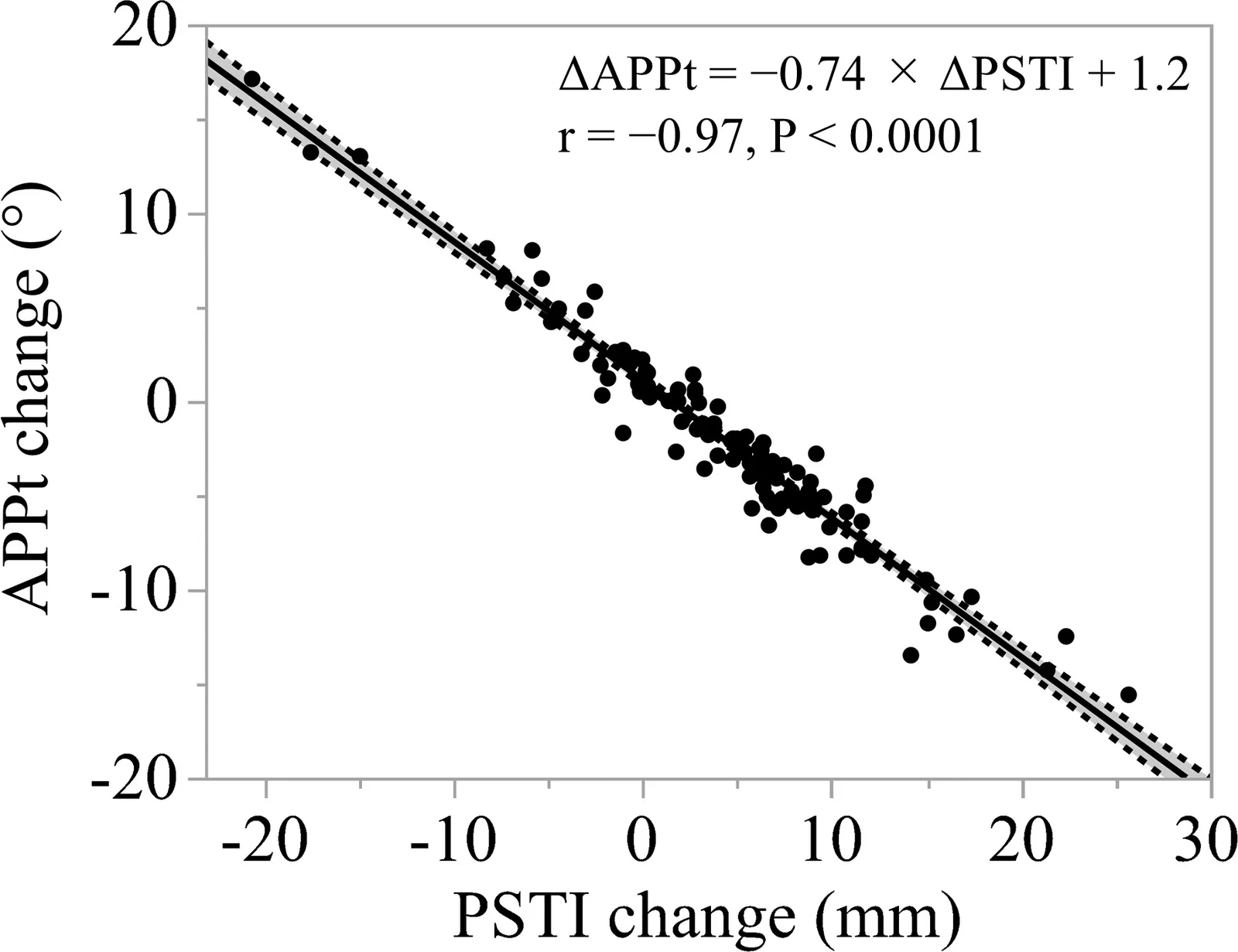
Relationship between changes in anterior pelvic plane tilt (ΔAPPt) and changes in pubic symphysis-to-transischial line distance (ΔPSTI) for the supine-to-standing transition. Scatter plot of ΔAPPt (°) measured on adjusted synthetic lateral radiographs versus ΔPSTI (mm) measured on clinical anteroposterior radiographs (standing minus supine). The solid line indicates the fitted linear regression, and the shaded band indicates the 95% CI.

Accuracy is summarized in Table III. In the derivation cohort, the mean error was −0.01°, with a RMSE of 1.3°, a MAE of 1.0°, and 95% LoA −2.6° to 2.5°.

**Table III.** Prediction accuracy of the regression model for estimating change in anterior pelvic plane tilt (APPt) and sub-group analyses. Prediction error was defined as change in anterior pelvic plane tilt (ΔAPPt) measured on adjusted synthetic (AS) radiographs minus predicted ΔAPPt. Within ± 3° and ± 5° indicate the number and percentage of cases in which the prediction error was within ± 3° and ± 5°, respectively. Sub-group analyses were performed in the independent temporal validation cohort, stratified by ΔAPPt measured on AS radiographs.

Analysis	Mean error (range), °	RMSE, °	MAE, °	LoA, °	Within ± 3°, n (%)	Within ± 5°, n (%)
Derivation cohort (apparent performance; n = 116)	−0.01 (−4.2 to 3.0)	1.3	1.0	−2.6 to 2.5	113 (97.4)	116 (100)
Fivefold cross-validation (n = 116)	0.01 (−4.3 to 3.4)	1.4	1.0	−2.6 to 2.7	112 (96.6)	116 (100)
Independent temporal validation (n = 80)	0.2 (−3.9 to 4.7)	1.3	0.9	−2.4 to 2.8	75 (93.8)	80 (100)
**Sub-group analyses**						
ΔAPPt < −10° (n = 7)	1.1 (−0.2 to 4.7)	1.9	1.2	−2.3 to 4.5	6 (85.7)	7 (100)
−10° ≤ ΔAPPt < −5° (n = 16)	−0.6 (−3.9 to 1.3)	1.7	1.2	−3.7 to 2.6	14 (87.5)	16 (100)
−5° ≤ ΔAPPt < 0° (n = 38)	0.2 (−2.1 to 3.4)	1.1	0.8	−1.9 to 2.3	37 (97.4)	38 (100)
ΔAPPt ≥ 0° (n = 19)	0.6 (−0.8 to 3.4)	1.2	0.8	−1.6 to 2.8	18 (94.7)	19 (100)

LoA, 95% limits of agreement; MAE, mean absolute error; RMSE, root mean square error.

Fivefold cross-validation yielded a mean error of 0.01°, a RMSE of 1.4°, a MAE of 1.0°, and 95% LoA −2.6° to 2.7°.

In addition, observer-specific prediction accuracy was consistent across observers in the 30-patient reliability subset, with RMSE values ranging from 1.4° to 1.7°, MAE values ranging from 1.0° to 1.4°, and all prediction errors falling within 5° of the observed ΔAPPt values (Supplementary Table S3).

In the independent temporal validation cohort (n = 80), performance remained clinically acceptable, with a mean error of 0.2°, a RMSE of 1.3°, a MAE of 0.9°, and 95% LoA −2.4° to 2.8°. Absolute error was ≤ 3° in 75 out of 80 (93.8%) cases and ≤ 5° in 80 out of 80 (100%) cases. Exploratory sub-group analyses stratified by ΔAPPt measured on AS radiographs showed consistent performance across strata (Table III).

In an exploratory sub-group analysis according to acetabular morphology, absolute APPt and PSTI values differed between patients with DDH and those with normal acetabula in the derivation cohort, whereas ΔAPPt and ΔPSTI did not differ significantly in either cohort. In the independent temporal validation cohort, prediction accuracy remained clinically acceptable in both acetabular morphology groups (Supplementary Table S4).

## Discussion

This study demonstrated that supine-to-standing within-patient changes in sagittal pelvic tilt (ΔAPPt) can be estimated from routine AP hip radiographs using changes in the pubic symphysis-to-transischial line distance (ΔPSTI). The derived regression equation (ΔAPPt = −0.74 × ΔPSTI + 1.2) showed clinically acceptable prediction accuracy, with 95% limits of agreement of approximately ± 3° in both fivefold cross-validation and independent temporal validation, and absolute error ≤ 5° in all cases in both validation procedures. Importantly, model development and validation were performed within the observed ΔAPPt range in this study (independent temporal validation cohort: −15.3° to 8.0°), and performance should be interpreted within this validated range and may not generalize beyond it.

The supplementary CT-based analysis supported the internal consistency of AS-measured APPt, although it did not establish criterion validity against an independent imaging reference standard. In the absence of routine same-session paired biplanar imaging, AS-measured APPt was used as a practical reference outcome for within-patient change.

Previous studies have shown that absolute pelvic tilt cannot be estimated accurately from AP radiographs because of anatomical variation. In contrast, within-patient changes in sagittal pelvic tilt, as well as related measurement parameters on AP radiographs, have demonstrated strong correlations with values obtained from cadaveric models, CT, and EOS imaging.^9-12^ These findings support a change-based framework rather than absolute tilt estimation when only AP radiographs are available.

Innmann et al^12^ reported that changes in pelvic tilt could be estimated from changes in the sacrofemoral–pubic angle using EOS images from standing to relaxed seated position. Their approach is conceptually important because it uses a coronal parameter to estimate sagittal pelvic tilt change. However, the equation was derived under EOS-based conditions and for a different postural transition. When applied exploratorily to our cohort, prediction errors were larger, supporting the need for protocol-specific derivation and validation (Supplementary Table S5).

A key distinction in our methodology was the use of the PSTI, a coronal parameter initially described by Muir et al.^10^ PSTI was measurable in all cases and demonstrated excellent intraobserver and interobserver reliability, with strong correlations between clinical and AS radiographs in both supine and standing positions. Linear regression showed a strong inverse association between ΔPSTI and ΔAPPt (R² = 0.94), indicating that ΔPSTI explained more than 90% of the variance in ΔAPPt. The model maintained high predictive accuracy without magnification adjustment based on femoral head diameter from preoperative CT. Although magnification adjustment was evaluated (ΔAPPt = −0.87 × ΔPSTI − 0.2), predictive performance was comparable with that of the unadjusted model, with only marginal differences in fivefold cross-validation (adjusted: RMSE 1.1°, MAE 0.9°; unadjusted: RMSE 1.4°, MAE 1.0°; Supplementary Table S6). These findings suggest that magnification adjustment offers limited additional benefit and that the simpler unadjusted model is adequate for clinical application. These features make ΔPSTI a practical coronal parameter for estimating within-patient sagittal pelvic tilt change from AP radiographs.

In the exploratory sub-group analysis according to acetabular morphology, absolute APPt and PSTI values differed between patients with DDH and those with normal acetabula, particularly in the derivation cohort, whereas ΔAPPt and ΔPSTI did not differ significantly between groups in either cohort. Prediction accuracy also remained clinically acceptable in both groups in the independent temporal validation cohort. These findings further support the use of a within-patient change-based approach, although validation in more diverse populations remains necessary.

Although the present equation was derived and validated for the supine-to-standing transition, it provides a simple, change-based method to quantify within-patient ΔAPPt from routine AP radiological measurements when paired supine and standing AP radiographs are available. Extension to other postural transitions or standing-only longitudinal assessment requires protocol-specific re-derivation and validation. Collectively, these findings support the use of this estimator for the supine-to-standing transition and provide a basis for future protocol-specific studies extending AP-only use to longitudinal and large-scale retrospective settings.

This study has some limitations. The proposed equation estimates within-patient changes in pelvic tilt rather than absolute pelvic tilt. Accurate assessment of absolute pelvic tilt would require imaging approaches that directly characterize pelvic orientation (e.g. EOS imaging or CT-based protocols), or paired biplanar radiographs acquired in the same posture and at the same time; however, such imaging is not routinely available in many clinical settings.

Model development and validation were performed within the observed ranges of ΔAPPt in this study (derivation cohort; −15.6° to 17.1°; independent temporal validation cohort; −15.3° to 8.0°). Accordingly, predictive performance outside these ranges, or in patients with more extreme pelvic tilt changes, remains uncertain and warrants further evaluation. In addition, criterion validity of APPt measured on AS radiographs against an independent imaging reference standard could not be established in our setting, because same-session paired biplanar radiographs in identical postures were not feasible.

Radiological acquisition variability may have affected measurement accuracy. Although the central beam was directed to the upper margin of the pubic symphysis, residual variation in beam position, film–object distance, and axial or coronal pelvic rotation could not be fully controlled.

This was a retrospective, single-institution study that included a relatively homogeneous Japanese patient population, with a high proportion of female patients and DDH cases, which may limit generalizability. Although exploratory sub-group analysis according to acetabular morphology showed clinically acceptable prediction accuracy in both the DDH and radiologically normal acetabulum groups, this analysis was not powered for sub-group comparisons. Although we selected coronal parameters supported by prior studies, not all potential coronal parameters were evaluated, which may limit the scope of our findings.^8-13^ External validation in more diverse populations and across institutions, including different imaging equipment and acquisition protocols, is needed to confirm robustness in broader clinical settings.

In conclusion, the proposed regression equation enables clinically acceptable estimation of within-patient sagittal pelvic tilt change from routine AP radiographs, with 95% LoA of approximately ± 3° within the validated range. These findings support a validated, change-based estimator for the supine-to-standing transition and provide a methodological foundation for future protocol-specific studies extending AP-based assessment to other settings.

**Take home message**

- A simple pubic symphysis-to-transischial line distance-based regression estimates within-patient supine-to-standing sagittal pelvic tilt change from routine anteroposterior hip radiographs.

- Accuracy is clinically acceptable, with 95% limits of agreement of approximately ± 3° in both fivefold cross-validation and independent temporal validation.

- Generalization to other transitions or standing-only follow-up requires protocol-specific re-derivation and validation.

## Data Availability

The data that support the findings for this study are available to other researchers from the corresponding author upon reasonable request.
